# Improved accessibility of emergency obstetrics and newborn care(EmONC) services for maternal and newborn health: a community based project

**DOI:** 10.1186/1471-2393-13-136

**Published:** 2013-06-24

**Authors:** Ali Turab, Shabina Ariff, Muhammad A Habib, Imran Ahmed, Masawar Hussain, Akhtar Rashid, Zahid Memon, Mohammad I Khan, Sajid Soofi, Zulfiqar A Bhutta

**Affiliations:** 1 Department of Paediatrics and Child Health, Division of Women and Child Health, Aga Khan University, Karachi, Pakistan; 2 Provincial Programme for Family planning and Primary health care (Lady Health workers programme) for Punjab, Lahore, Pakistan; 3 Maternal and Newborn Health Programme, Research and Advocacy Fund, Islamabad, Pakistan; 4 International Vaccine Institute, Seoul, Korea; 5 Founding Director Center of Excellence in Women and Child Health, Aga Khan University, Karachi, Pakistan; 6 Co-Director, Center for Global Child Health, Hospital for Sick Children, Toronto, Canada

**Keywords:** Emergency Obstetrics and Newborn Care (EmONC), Perinatal mortality, Neonatal mortality, Reproductive health, Child health, Pakistan

## Abstract

**Background:**

Every year an estimated three million neonates die globally and two hundred thousand of these deaths occur in Pakistan. Majority of these neonates die in rural areas of underdeveloped countries from preventable causes (infections, complications related to low birth weight and prematurity). Similarly about three hundred thousand mother died in 2010 and Pakistan is among ten countries where sixty percent burden of these deaths is concentrated. Maternal and neonatal mortality remain to be unacceptably high in Pakistan especially in rural areas where more than half of births occur.

**Method/Design:**

This community based cluster randomized controlled trial will evaluate the impact of an Emergency Obstetric and Newborn Care (EmONC) package in the intervention arm compared to standard of care in control arm. Perinatal and neonatal mortality are primary outcome measure for this trial. The trial will be implemented in 20 clusters (Union councils) of District Rahimyar Khan, Pakistan. The EmONC package consists of provision of maternal and neonatal health pack (clean delivery kit, emollient, chlorhexidine) for safe motherhood and newborn wellbeing and training of community level and facility based health care providers with emphasis on referral of complicated cases to nearest public health facilities and community mobilization.

**Discussion:**

Even though there is substantial evidence in support of effectiveness of various health interventions for improving maternal, neonatal and child health. Reduction in perinatal and neonatal mortality remains a big challenge in resource constrained and diverse countries like Pakistan and achieving MDG 4 and 5 appears to be a distant reality. A comprehensive package of community based low cost interventions along the continuum of care tailored according to the socio cultural environment coupled with existing health force capacity building may result in improving the maternal and neonatal outcomes.

The findings of this proposed community based trial will provide sufficient evidence on feasibility, acceptability and effectiveness to the policy makers for replicating and scaling up the interventions within the health system

**Trial registration:**

ClinicalTrial.gov NCT01751945

## Background

### Global neonatal and maternal mortality and in Pakistan

Globally over three million neonates die every year [1]. Overall under-5 mortality over the past decade has been significantly curtailed however the burden of neonatal deaths is more or less the same and these now represent about 40% of all deaths in children under the age of 5 [1,2]. Majority of these neonates die in rural areas of underdeveloped countries and approximately two thirds are due to infection and complications related to low birth weight (LBW) and prematurity [1-3]. Over 287,000 mothers died in 2010 worldwide and almost 60% of total maternal death burden is concentrated in 10 countries and Pakistan is one of them with 12000 annual deaths [4]. Postpartum hemorrhage is the leading cause of maternal death whereas infections (maternal puerperal sepsis) are the third most frequent cause of mortality globally accounting for over 12% of all maternal deaths [5,6].

Pakistan has relatively worse neonatal and maternal health indicators than its neighboring countries in south Asia [7,8]. According to recent UNICEF estimates, the country has a neonatal (NMR) and under 5 year mortality rates of 36 and 72 per 1,000 live births and maternal mortality ratio (MMR) of 280 per 100,000 live births respectively [7]. Over 200,000 newborns die each year which represent 58% of total under 5 child deaths in the country. In Pakistan, majority of mothers (74%) living in rural areas deliver at home and the risk of neonatal death is more marked in rural (55/1000 live births) areas compared to urban settings (48/1000 live births) [8].

### Health care infrastructure in Pakistan

Pakistan has an impressive infrastructure for primary health care with a network of basic health units (BHU) and rural health centers (RHC) [9]. While the RHCs and BHUs are staffed by qualified medical and nursing staff, services for basic or comprehensive emergency obstetric and newborn care (EmONC) at the level of BHUs and RHCs are not always of highest quality nor are they accessible [9]. Health care at the community level is largely supported by a large number of trained cadres of community health workers (CHW). In Pakistan, one of the major CHW programs is the lady health worker (LHW) program. Currently almost 102,000 LHWs are in place and they receive basic training in maternal and newborn care and provide this care in homes in the communities in which they work [8]. Although they are not directly involved in conducting deliveries, these LHWs are supposed to function in close liaison with BHU and RHC staff in providing antenatal care, contraceptive advice, growth monitoring, and immunization services. The ministry of health has deployed a new cadre of CHW; community midwives (CMW’s) to phase out the traditional birth attendants (TBA’s). While CMWs are trained in the identification of complicated pregnancy and its management, but very few have been deployed so far. Thus, in order to improve maternal and newborn care in Pakistan there is considerable potential for training of LHWs, CMW’s and TBA on basic and comprehensive Emergency Obstetrics and Newborn Care (EmONC).

### Rationale and contents of EmONC package

The proposed EmONC package consists of maternal and neonatal health pack, enhanced trainings of community based health care providers and community mobilization. These evidence based interventions are proven to reduce incidence of neonatal morbidity and mortality as well as to provide early identification of danger signs so that early intervention can be made. The maternal and neonatal health pack includes 4% chlorhexidine (CHX) for application to the umbilical cord. It is known to prevent neonatal omphalitis by 75% and neonatal mortality by 25-40% [10-12]. It also contains emollient (sunflower oil) which promotes skin integrity and reduces portal of entry for infection and prevents hypothermia. In hospitalized preterm infants it has proven effectiveness of 26% reduction in mortality [13]. Additionally the pack will contain clean delivery kit (CDK) which is associated with lesser incidence of omphlitis and puperal sepsis [14-17].

The EmONC package also encompasses ‘enhanced trainings’ and ‘community mobilization’ interventions to address the delays for accessing quality EmONC services. These interventions have proven effectiveness in reducing neonatal and maternal mortality [18]. The LHW’s of the intervention arm shall be provided with neonatal weighing scales for recognition of low birth weight (LBW) and prompt referral of such cases to the health system to reduce associated neonatal morbidity and mortality.

There is considerable evidence on independent effectiveness of interventions included in the package. However, the combined impact of these interventions together on maternal and newborn mortality has not been demonstrated in any trial. The results of our study will provide important information for policy makers and health managers in addressing maternal and newborn health collectively.

### Hypothesis

We hypothesize that provision of this integrated EmONC package consisting of evidence based interventions, trainings and community mobilization will reduce perinatal and all cause NMR by up to 20%. We expect that this package will reduce all cause NMR through a reduction in both infectious causes of death and low birth weight (LBW).

## Methods/Design

### Study design

**Figure 1:**
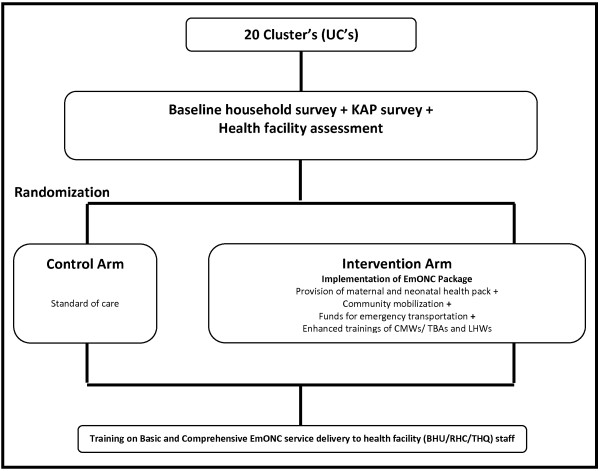
**Schema of activities and interventions to be done as part of the trial.** UC’s = Union councils, KAP = Knowledge, Attitude and Practices, EmONC = Emergency Obstetrical and Newborn Care, CMW = Community Midwife, TBA = Traditional Birth Attendant, LHW = Lady Health Worker, BHU = Basic Health Unit, RHC = Rural Health Center, THQ = Tehsil Headquarter Hospital.

This community based cluster randomized controlled trial (c RCT) will evaluate the impact of an EmONC package in the intervention arm compared to standard of care in control arm. Figure 1 describes the schema of intervention and activities to be done as part of the trial.

### Study outcome measures

The primary outcome of the study is reduction in perinatal and neonatal mortality. Perinatal mortality is defined as the composite rate of stillbirth and 7-day neonatal mortality per 1000 live births per year.

### Sample size

We assume that the average cluster size is 15000 population, considering a crude annual birth rate of 20 per 1000 population and estimated average perinatal mortality rate of 60/1000 with a coefficient of variation (k) between clusters of 0.125, an intracluster correlation coefficient of 0.05 and to detect a 20% difference in the mortality rates between intervention and control clusters, we would require at least 20 clusters, ten clusters per arm to provide the study with a power of 90% over a three-year intervention period.

### Study setting

**Figure 2:**
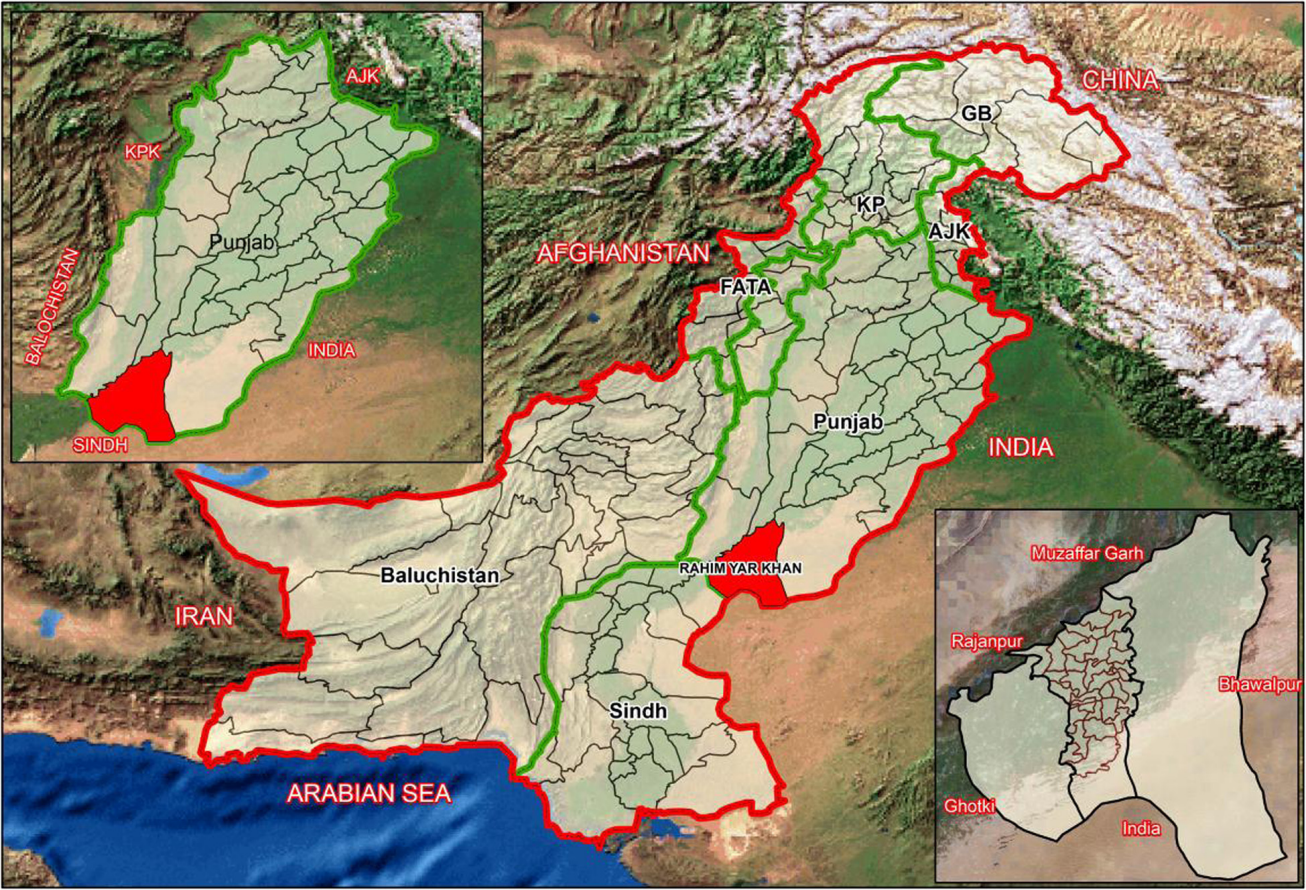
**Map of Pakistan showing study area in District Rahimyar Khan, Province Punjab.** International Boundary, Province Boundary, District Boundary, Tehsil Boundary, UC Boundary.

The study will be conducted in District Rahimyar Khan (RYK) of Punjab province (Figure 2). Rahimyar Khan is predominantly a rural district. Administratively the districted is sub-divided into Sadiqabad, Rahimyar Khan, Khanpur and Liaqatpur ‘Tehsils’. The study shall be implemented in Tehsil Rahimyar Khan. The Tehsil is further sub-divided into 40 Union Councils (UC’s). These small geographical subdivisions defined for administrative purposes. Each UC serves ~15000 - 20000 population and usually contains a Basic Health Unit (BHU) or a Rural Health Center (RHC) which provides basic EmONC facilities. Catchment population of single UC along with linked BHU and RHC shall be considered as one cluster and 20 UC’s (clusters) shall be selected for the implementation of the trial.

### Study population

Our target population for the intervention arm will include all currently pregnant women (15–49 years) or those who will conceive during the course of study and their newborns in these 20 UC’s. Regardless of their age and socio-medical profile they will be offered the services, as per the trial protocol. The women in the control arm shall receive standard care through the existing health system.

### Baseline data collection

A quantitative research method approach shall be followed to collect baseline information related to the project outcome. The results of baseline will be compared with subsequent outcome surveillance findings for impact assessment of the interventions on outcome measures.

#### Quantitative approach

##### Baseline household cross sectional survey

A door to door household cross sectional survey will be conducted in the study area to collect information on socio-demographic characteristics, maternal and neonatal morbidity and mortality. The data collection teams shall seek verbal consent and explain the purpose of visit before the start of interview.

##### Survey of knowledge, attitude and practices (KAP)

Additionally, to assess the knowledge, attitude and practices of communities regarding maternal and newborn health problems and care seeking patterns in the study area another survey nested within baseline cross sectional household survey shall be conducted in a smaller representative sample of the study population. A random list of household with a mother who delivered in the last one year shall be generated from household survey data. Prior to start of interview data collector’s verbal consent shall be sought and purpose of visit explained.

##### Health facility assessment

A rapid census of health care providers and primary health care facilities will be carried out in the study area as a part of baseline activity. The purpose of this survey shall be to map out the facilities offering maternal, newborn and child health services and to describe the distribution, availability, functioning and quality of services for maternal, neonatal and child health (MNCH). This exercise would help to plan the training activities and modules according to the capacities and service delivery related to obstetric and newborn care.

### Randomization

Blocked randomization of clusters will be done after stratifying the clusters based on information collected from the baseline survey. The strata will consider the number of the targeted population in the cluster, maternal and infant mortality, access to health centers and the number of health workers. Due to the nature of the intervention blinding is not possible, however to ensure reduced measurement bias data on the effect of the intervention will be collected by separate teams.

### Intervention (emonc) package

As discussed in the background a comprehensive package of low cost community interventions is developed on the basis of available global and local literature around maternal and neonatal care and survival. EmONC package is composed of a maternal and neonatal health pack, enhanced trainings of health workers and community mobilization.

#### Maternal and neonatal health pack

A pack consisting of 4% CHX solution, emollient (sunflower seed oil) and clean delivery kit CDK and health messages brochure will be delivered to expectant mothers of intervention arm in third trimester of pregnancy of intervention arm.

##### CHX

It will be dispensed as 4% solution in 15 ml dispensing bottles to be applied on umbilical cord once daily up to 10 days starting from the first day of life.

##### Emollient (sunflower seed oil)

Additionally the pack shall contain 50 ml of emollient (sunflower seed oil) in dispensing bottles for newborn massage. The mother will be advised to massage their newborns once a day from third day of life up to twenty eighth day.

##### CDK

Contents of CDK will include a hotel-size bar of soap for the birth attendant to wash hands both before delivery and prior to cutting the umbilical cord, and to clean the mother’s perineum. The CDK will also have a pair of clean disposable gloves for reducing disease transmission, a square yard of clean plastic sheeting for provision of a clean surface to deliver the baby and one clean single use razor blade for clean cutting of the cord and a sterile thread and cord clamp.

##### Health messages brochure

Along with the pack expectant women will be handed over a brochure containing messages on birth preparedness, awareness and recognition of danger signs in mothers and newborns, acute obstetric and neonatal emergencies and information on immediate care of newborn (prevention of hypothermia in low birth weight babies, immunization and exclusive breast feeding etc.).

#### Maternal and neonatal health pack delivery

The intervention pack shall be delivered to the expectant women at the household level by the area Lady Health Workers (LHW’s). Apart from their routine assignment these LHW’s will visit the households of the expectant women during the (third trimester of pregnancy) in the intervention arm and hand over the health pack to the family/expectant mother. During the visit the LHW will explain the health education messages (health messages brochure) focused on importance of antenatal, natal and postnatal care along with instructions on how to use the pack.

#### Equipping ‘the lady health worker’s (lhw)’ and ‘community midwife’s (CMW)’

The LHW’s and CMW’s of the intervention arm shall be provided with neonatal and adult weighing scales to record mother’s weight during antenatal visits and newborn’s weight at birth or soon after birth. Objective of this activity is recognition of low birth weight (LBW), prompt provision of care of low birth babies and subsequent referral if required at the health system level to reduce associated neonatal morbidity and mortality.

Moreover the LHW’s of the intervention arm will be provided with Amoxicillin drops, to be administered to newborn and infants with suspected sepsis/pneumonia as a first dose (domiciliary care) prior to referral to the nearest health facility for further management.

#### Enhanced trainings

The LHWs, CMWs and traditional birth attendants (TBAs) of the intervention arm will receive trainings on basic obstetric and newborn care. They will also be trained on recognition of danger signs and early referrals. Similarly the physicians, LHV and nurses of primary health care facilities (BHU, RHC) will receive training in basic emergency obstetric care (EmOC), essential newborn care (ENC) and neonatal resuscitation in both intervention and control arms. Where infrastructure will allow the Physicians, LHV/Nurses will also receive training on comprehensive EmONC.

#### Community mobilization

##### Health promotion

To create awareness and promote maternal, neonatal and child health (MNCH) in the community at household level, women and their husbands will receive awareness on maternal and neonatal health in the intervention clusters. The health sessions will be facilitated by the area LHW. These sessions will be aimed to deliver awareness on antenatal care, birth preparedness; essential and immediate newborn care and recognition of danger signs, management low birth weight and sepsis with early and appropriate referral. An emergency fund will also be established with the help of area LHW for active referral and transportation of high risk and complicated cases. This fund shall be established through the local resources and families of expectant women.

##### Heath sms

Standard health SMS on MNCH will be designed and subsequently delivered to create awareness amongst expectant or delivered women of reproductive age and their families regarding antenatal, natal and post natal care, and general health. These messages will be delivered to the women’s cell number or her family members throughout the pregnancy, childbirth and post-partum period in the intervention arm.

### Quarterly surveillance of outcome measures

A surveillance system will be instituted in the intervention and control arms. Each surveillance round shall span across three months and every household in the study area will be visited once. This activity will be conducted by a team of data collectors who will work independent to LHW’s who will be only responsible for delivering the ‘maternal and neonatal health pack’. Information will be collected on demographics, pregnancy outcomes, health seeking patterns and maternal and neonatal outcomes in context to major morbidities and mortalities. Through surveillance, information will also be gathered on the impact of the interventions in target population. The results of surveillance rounds will be compared with baseline findings to assess the impact of EmONC package on the outcomes measures.

### Data management

Pretested standard and structured questionnaire shall be developed for baseline and KAP surveys and quarterly surveillance data collection. The data collectors shall receive classroom and real life scenario training on the questionnaires. Each form will have an individual study identification number to ensure confidentiality and linkage.

Data on outcome and process indicators will be collected separately for the trial. Outcome data on perinatal and neonatal mortality and health care utilization will be collected through quarterly surveillance system using standardized instruments. Data collection teams shall visit the households with pregnant/delivered women every three months. A structured questionnaire asking information on antenatal, natal and postnatal care practices, morbidities and mortalities will be administered to these women. Women who deliver in hospital or in another house will be revisited.

The trial implementation data (process data) will also be collected on standardized forms; process data will include information on team and trainings activities. Inventory record for the delivery and usage of every maternal and newborn health pack shall be maintained by the data collection teams. Additional logs for distribution of Amoxil to the sick child, measurement of birth weight and referral shall be obtained by the LHW supervisor on weekly basis.

All forms filled shall be checked by supervisors for completeness and consistency, and then transferred to the Data Management Unit (DMU). Data shall be dual entered onsite in real time by trained data input operators in the customizable Visual FoxPro database. Consistency, validity, and referential integrity shall be enforced through automated post-entry checks on the database. Collected data will be retained in a secure location at the site office.

### Data analysis plan

Primary outcome of interest (perinatal and neonatal mortality) will be measured through household surveillance activities during the follow up. Surveillance will be carried out in both intervention and control arms. To capture all birth and death, each household will be visited quarterly during the course of the study. This method will ensure reduction in recall bias with other measures of association with primary outcome of the study.

Two approaches will be followed to assess the impact of the intervention on outcome. Following descriptive analysis for assessment of distribution of various factors within and between groups, we will compare the baseline rates between the two arms of the study, while adjusting for confounding factors, and then do the same at the end of the trial. Cluster adjusted poison regression analysis will be performed to compare maternal and newborn mortality rates. In addition, we will also assess the trends in mortality using the follow up surveillance data for KAP and maternal and newborn health service utilization. We hypothesize that the KAP trends would not be different at baseline; however with intervention being effective will change over time.

### Ethical considerations

Informed verbal consent will be sought from representatives of all participating communities involved in the study prior to implementation. The consent will explicitly outline the aims and objectives of the study along with the strict confidentiality of provided information. For data collection purposes, informed consent will also be sought from all pregnant women that are residents of the study area.

The Ethics Review Committee (ERC) of Aga Khan University has granted approval to the proposed trial (Ref No. 2146-Ped-ERC-12). In addition National Bioethics Committee (NBC) of Pakistan has also approved the study for human subject research (Ref No. 4-87/12/NBC-84/RDC/2031

## Discussion

In resource poor settings, many mother delivers at home and receive no specific medical care. In these areas, geography, infrastructure, and poverty often effectively prevent access to health centers and care. Home outreach with trained CHWs is increasingly recognized as the mainstay for provision of maternal and newborn care in these settings. Apart from direct provision of interventions, research has shown that interventions that involve community mobilization and counseling sessions for mothers on topics related to birth and newborn care preparedness for bringing behavior change can reduce total neonatal deaths by 21% and early neonatal deaths by 24% (18). On the other hand, improvement of women’s access to antenatal, intrapartum and postnatal care with training cadre of community workers, and traditional birth attendant can reduce maternal morbidities by 25% and further improve total neonatal mortalities by 23% (18). However, these services are not always available to those who need them most nor have they been packaged into a single consolidate program. Such a package consisting of low cost, evidence based interventions for use in the home the home has tremendous potential to improve health status and decrease MMR and NMR.

Even though there is substantial evidence in support of effectiveness of various health interventions for improving maternal and child health, reduction in perinatal and neonatal mortality remain a big challenge for improving maternal and child health in a resource constrained and diverse country like Pakistan and achieving MDG-4 appears to be a distant reality. A comprehensive package of community based low cost interventions along the continuum of care tailored according to the socio cultural environment of the country along with capacity building may improve the maternal and neonatal outcomes.

The findings of this critical community based trial will provide sufficient evidence on the implementation of the intervention and its impact to the policy makers, for replicating and scaling up sustainable interventions in rural settings.

## Competing interest

The authors of this manuscript declare that they have no competing interest (financial or otherwise) in this publication.

## Author’s contribution

SS conceived the idea, study design and as principal investigator was involved in all aspects of this study. ZAB provided the technical and intellectual inputs. SA, AT, MAH, IA, ZM, MIK, ZSL, AR and MH were involved in study design, implementation, analysis and planning of the study. AT produced the first draft and subsequent drafts of the paper. ZAB is the guarantor of the study. All authors reviewed and approved various drafts and the final paper.

## Pre-publication history

The pre-publication history for this paper can be accessed here:

http://www.biomedcentral.com/1471-2393/13/136/prepub
